# Belly fat or bloating? New insights into the physical appearance of St Anthony of Padua

**DOI:** 10.1371/journal.pone.0260505

**Published:** 2021-12-21

**Authors:** Jessica Mongillo, Giulia Vescovo, Barbara Bramanti

**Affiliations:** Department of Environmental and Prevention Sciences, University of Ferrara c.so Ercole I d’Este n.32, Ferrara, Italy; The Cyprus Institute, CYPRUS

## Abstract

Over the centuries, iconographic representations of St Anthony of Padua, one of the most revered saints in the Catholic world, have been inspired by literary sources, which described the Saint as either naturally corpulent or with a swollen abdomen due to dropsy (i.e. fluid accumulation in the body cavities). Even recent attempts to reconstruct the face of the Saint have yielded discordant results regarding his outward appearance. To address questions about the real appearance of St Anthony, we applied body mass estimation equations to the osteometric measurements taken in 1981, during the public recognition of the Saint’s skeletal remains. Both the biomechanical and the morphometric approach were employed to solve some intrinsic limitations in the equations for body mass estimation from skeletal remains. The estimated body mass was used to assess the physique of the Saint with the body mass index. The outcomes of this investigation reveal interesting information about the body type of the Saint throughout his lifetime.

## Introduction

**Fig 1:**
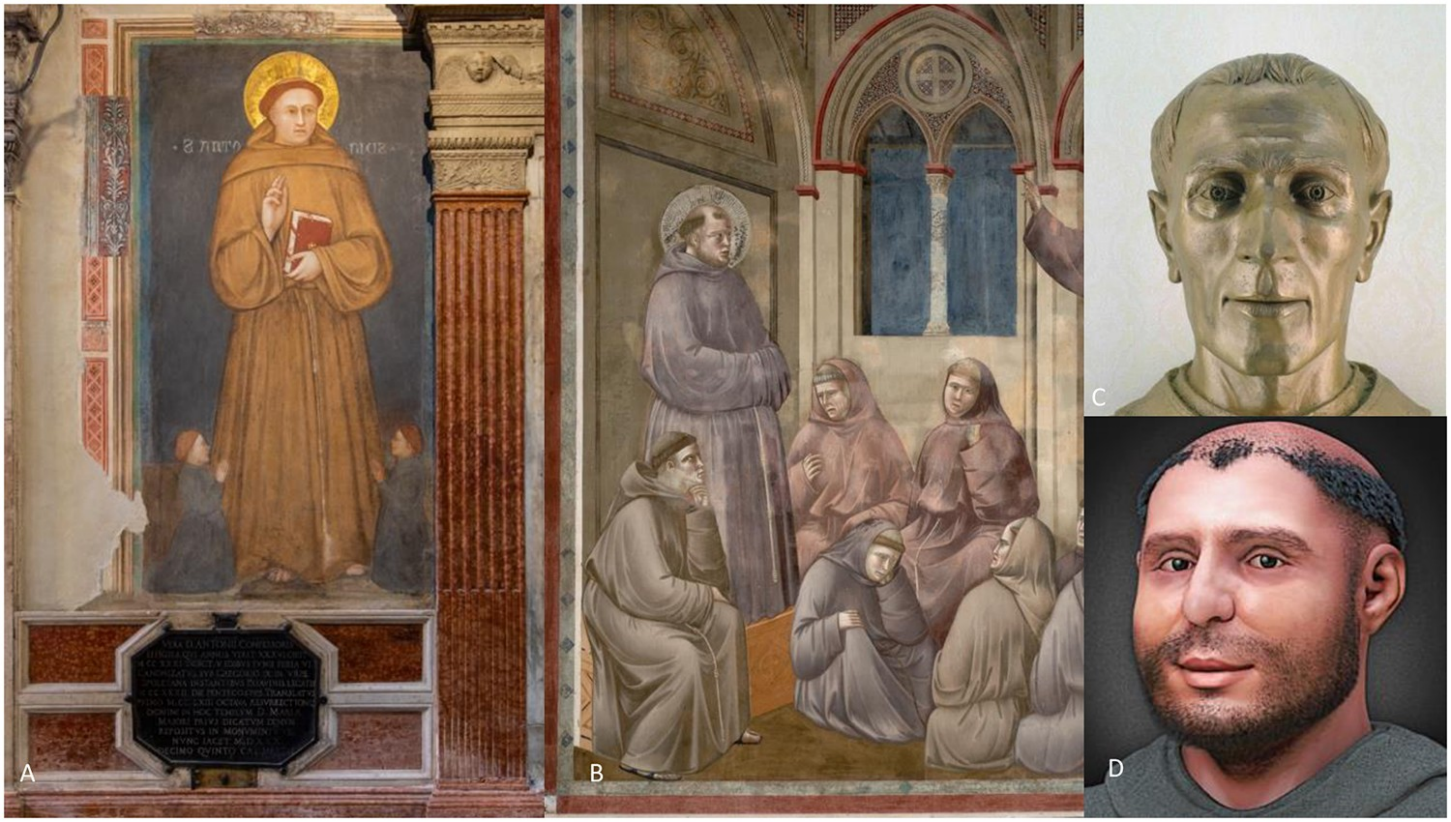
The representation of St Anthony. (A) St Anthony giving his blessing, Giotto School. This portrait is considered to reflect the true effigy of the Saint. Padova, Basilica del Santo (1238–1310), Giovanni Pinton/ Archivio Fotografico Messaggero di Sant’Antonio, 2020. (B) Giotto, St Francis appears in the Chapter of Arles, ca. 1295–1299. Assisi, Chiesa Superiore of Basilica di San Francesco. In the detail, St Anthony is illustrated suffering, with a bloated abdomen. (C) Bust of St Anthony, detail. Bronze sculpture by Roberto Cremesini, 1995. This is a scientific reconstruction of the ’real’ face of the Saint, based on the skull found after the recognition of his body in 1981. In the reproduction of this bust, the artist relied on the advice of three scholars: C. Corrain (anthropologist), V. Meneghelli (anatomist) and V. Terribile Wiel Marin (anatomopathologist). Photo by Giorgio Deganello/ Archivio Fotografico Messaggero di Sant’Antonio, 1995. (D) The 3D Forensic Facial Reconstruction of St Anthony of Padua. Cicero Moraes—Opera propria, CC BY 3.0, https://commons.wikimedia.org/w/index.php?curid=33660858.

St Anthony of Padua (born Ferdinando Buglione, Lisbon 1195- Arcella, Padua 1231), is a saint venerated by the Catholic Church. He is very popular in Italy as well as being the Patron Saint of Brazil, Portugal and of the Custody of the Holy Land. The cult of St Anthony spread rapidly in the Mediterranean Catholic world during his lifetime due to his reputation as a thaumaturge and expanded globally in the 16^th^ and 17^th^ centuries thanks to the Portuguese cultural influence. In 1946, he was proclaimed a Doctor of the Universal Church [1]. Apart from the devotional aspect, several famous painters and sculptors have tried to propose a historically credible portrait of the Saint. The artists made different use of the information derived from hagiographic sources. The representation of St Anthony by Giotto School (1238–1310) (Fig 1A) seems to be inspired by the opus *Vita prima o Assidua* (1232, by Anonymous, this work represents one of the most important hagiographic sources), which described St Anthony in the last months of his life as endowed with a “natural corpulence” (*Vita Prima*, XI,7, p. 159) [2]. However, in the Legenda *Raymondina*, attributed to the Italian Franciscan Pietro Raimondi and dated around 1293 [3] the term ‘dropsy’ was firstly reported referring to the conditions of the Saint at the end of his life (“*Cum enim esset naturali corpulentia gravis et hydropisi etiam laboret*…” “Despite being severely corpulent by nature, and also afflicted with dropsy…”) (Raymundina, IX, 5, p. 26) [4]. In the Assisi’s frescos, dated to the years 1292–1296 (Fig 1B), Giotto portrayed St Anthony in a suffering condition, with a bloated belly, which possibly recalls a “dropsy”.

In ancient times, the term ‘dropsy’ was commonly used to describe generalized abdominal swelling (oedema or fluid retention), a symptom that is often associated with heart failure [5]. The written sources closest in time to the life of the Saint (*Vita Prima* written immediately after the death of St Anthony and *Legenda Raymondina*, written around 60 years later) seem to report both corpulence and dropsy as two distinct conditions. Both conditions have influenced all the subsequent representations and descriptions of the Saint. Indeed, the hagiography proposed by the Abbot Emanuelle De Azevedo (1832) described the Saint as short in stature, well-nourished and puffy with a round face, lively eyes, a high forehead, a beautiful, affable, and cheerful physiognomy, whereas dropsy is proposed as the cause of his bloated abdomen [6].

In 1981, in the context of the public ostension of the skeletal remains of the Saint, classical anthropological analyses and osteometric measurements were carried out by Cleto Corrain to answer some key questions about his life history. On this occasion, a cast of the skull was made, which allowed the sculptor Roberto Cremesini to attempt a more scientific reconstruction of the face of St Anthony. The Saint was represented with an oblong face and hollow eyes (Fig 1C), in agreement with the description of another literary source, the *Sancti Antonii confessoris de Padua vita* of Sicco Ricci Polentone (c. 1435) [7]. This is the only text that proposes a portrait of St Anthony with the same characteristics as in Cremesini’s work. In 2014, from the same cast, a 3D cranio-facial reconstruction was proposed, using both anthropological data and information from historical written sources [8]. To generate a more realistic face, the depth of the soft tissues was chosen from tables for an overweight body mass index (BMI>25) [9], although no attempt was done before to directly calculate the BMI from the skeletal remains [8] (Fig 1D).

Using osteometric measurements taken during the body recognition in 1981 [10], we applied estimation formulae to calculate St Anthony’s actual body mass (BM), and to address the question whether he was “naturally” overweight or not. Body mass estimation from skeletal remains relied on two approaches: the morphometric and the mechanical approach [11]. The morphometric method is based on the relationships between stature and selected body breadth measures [12]. In particular, the combination of stature and bi-iliac breadth (ST/BIB) has been frequently used on skeletal remains [13–19]. By contrast, the mechanical approach is built on the dimensions of the skeletal elements that mechanically support the weight of the body [11]. Femoral head breadth (FHB) has been mostly used for this purpose [11, 12, 20–29]. Mechanical methods are the most widely used, as they take in account several dimensions of different articular surfaces such as that of the knee, but also diaphysis breadths, and cross-sections of the bones [30–32]. Incidentally, these are the anatomical elements that are most preserved in the archaeological record. In addition, several studies focus on the relationship between weight and degenerative joint disease to improve understanding of the effect of the weight on joints [33–38].

We calculated the Body Mass Index (BMI), developed by Quetelet in 1832, and known as BMI since 1972 due to the work of Keys et al. (1972) [39, 40], to categorize the weight condition of the Saint. The BMI classification employed by the World Health Organization (WHO) and by the Center of Disease Control and Prevention (CDC) is useful to classify individuals according to weight and height and assess the health risk associated with weight [41].

Despite the difference in activity and adiposity levels between past and present populations, the BM formulas and the BMI can be employed in both bio-archaeological and forensic contexts as a valuable procedure to implement the biological profile of an individual.

## Materials and methods

The osteometric measurements of the bi-iliac breadth, femoral head breadth, femoral bi-epicondylar breadth and breadth of the tibial plateau were taken from published data [10]. In 1981, Cleto Corrain carried out the osteometric recognition on occasion of the public ostension of St Anthony’s skeletal remains.

We estimated the body mass of St Anthony according to several methods previously published (Table 1):

1. Femoral head body mass estimation (FHB-1), a sex-specific method [12]
2. Femoral head body mass estimation (FHB-2) [22]
3. Femoral head body mass estimation (FHB-3) [20]
4. Femoral head body mass estimation (FHB-4) [42]
5. Femoral head body mass estimation (FHB-5), combined with the femoral maximum length (FXL) [18]
6. Knee measurements: femoral epicondylar breadth (FBEB) and medio-lateral breadth of the tibial plateau (TPML) [43]
7. The stature/bi-iliac method 1 (ST/BIB-1) [14]
8. The stature/bi-iliac method 2 (ST/BIB-2) [18]

**Table 1:** Regression equations of body mass estimation that were used in this study, and their SEE (standard error of the equations, as reported in the quoted studies).

*Authors*	*Equations*	*SEE*
*Ruff et al., 1991*	BM = 2.741 * FHB-1 − 5.9	13.7
*McHenry, 1992*	BM = 2.239 * FHB-2 − 39.9	0.033
*Grine, 1995*	BM = 2.268 * FHB-3 − 36.5	4.3
*Ruff et al., 2012*	BM = 2.80 * FHB-4 − 66.7	6.8
*Niskanen et al., 2017 (1)*	BM = 0.600 * FHB-5 + 0.206 * FXL − 56.536	6.7
*Niskanen et al., 2017 (2)*	BM = 0.467 * ST-2 + 3.761 * LBIB − 119.537	8.93
*Ruff et al., 2005*	BM = 0.422 * ST-1 +3.126 * BIB − 92.9	3.7
*Keisu et al., 2019 (1)*	BM = 1.07 * FBEB − 15.88	10.6
*Keisu et al., 2019 (2)*	BM = 1.25 * TPML − 22.75	10.7

We estimated the stature from the skeleton according to Trotter & Gleser (1958) [44], using the average of the maximum lengths of the left femur, tibia, fibula, humerus and the right radius and ulna (measurements of the left radius and ulna were not recorded, since the bones were exhibited as a relic elsewhere). The employment of these equations appears to be appropriate, as shown by the calculation of the crural index for St Anthony (81.2), which is very similar to that obtained by Trotter & Gleser (1958) [44] for male individuals (81.9).

Following the advice of Elliott et al. (2016) [17] and the indications of Auerbach & Ruff (2012) [11], we calculated the average of three FHB estimates (1;2;3). Averaging the three FHB equations is the best approach when an individual does not fit one of the equations, which are targeted at small body-size [22], large body-size [20], and modern populations [12], respectively.

The measurement of the skeletal bi-iliac breadth was transformed in an assessment of the living bi-iliac breadth following the formula of Ruff (1997) [45], *Living = Skeletal x 1*.*17–3 (cm)*. In order to obtain information about the nutritional status of St Anthony, the body mass index, $BMI(\frac{kg}{m^{2}})$, was calculated as well, using the estimations of BM. Following the suggestions of WHO and CDC, a BMI value below 18.5 indicates underweight conditions; BMIs between 18.5 and 24.9 indicates normal weight conditions; overweight conditions is considered if the BMI is 25–29.9, and obese if it is a BMI greater than 30.

## Results

The estimation of the stature, obtained with the method of Trotter & Gleser (1958) [44] reveals a value of 171.1± 3.23 cm, which is similar to the stature defined by Cleto Corrain averaging the estimations obtained from all long bones (172.1 cm), independently of the laterality. However, Corrain attempted other assessments to conclude that St Anthony’s stature was likely 170 cm. Taking into account his conclusion and considering that this stature is included within the range we have calculated, we decided to use this value for the calculation of the BMI.

Table 2 shows the BM estimation values (kg) and the BMIs obtained from the different regression equations. In general, all formulae of the biomechanical method yielded lower values, with the FHB-4 formula [42] providing the smallest value (60.8±6.8). The average of the three combined formulae (FHB-1-2-3) indicates low value of body mass as well (66.2±6.0).

**Table 2:** Body mass and body mass index of St Anthony of Padua.

*Authors*	*Application*	*BM (kg)*	*SEE*	*BMI*	*SD*
*Ruff et al., 1991*	BM = 2.741 * 45.5 − 5.9	66.2	6.0	22.9	± 2.1
*McHenry, 1992*	BM = 2.239 * 45.5 − 39.9				
*Grine, 1995*	BM = 2.268 * 45.5 − 36.5				
*Ruff et al., 2012*	BM = 2.80 * 45.5 − 66.7	60.7	6.8	21.0	± 2.3
*Niskanen et al., 2017 (1)*	BM = 0.600 * 45.5 + 0.206 * 471 − 56.536	67.8	6.7	23.5	± 3.0
*Niskanen et al., 2017(2)*	BM = 0.467 * 171.1 + 3.761 * 30.93 − 119.537	76.2	8.93	26.3	± 1.3
*Ruff et al., 2005*	BM = 0.422 * 171.1 +3.126 * 30.93 − 92.9	75.5	3.7	26.1	± 3.6
*Keisu et al., 2019(1)*	BM = 1.07 * 86 − 15.88	76.1	10.6	26.3	± 3.7
*Keisu et al., 2019 (2)*	BM = 1.25 * 81 − 22.75	78.5	10.7	27.1	± 2.1

SEE (standard error of equations, as reported in the quoted publications)

The combination of stature and living bi-iliac breadth (morphometric method) yielded the highest values both applying the formula of Ruff et al. (2005) [14] (75.5±3.7), and that of Niskanen et al. (2017) [18] (76.0±3.6). Similar values resulted from the equation based on the knee joint dimensions of Keisu et al. (2019) [43] (76.1±10.6; 78.5±10.7).

**Fig 2:**
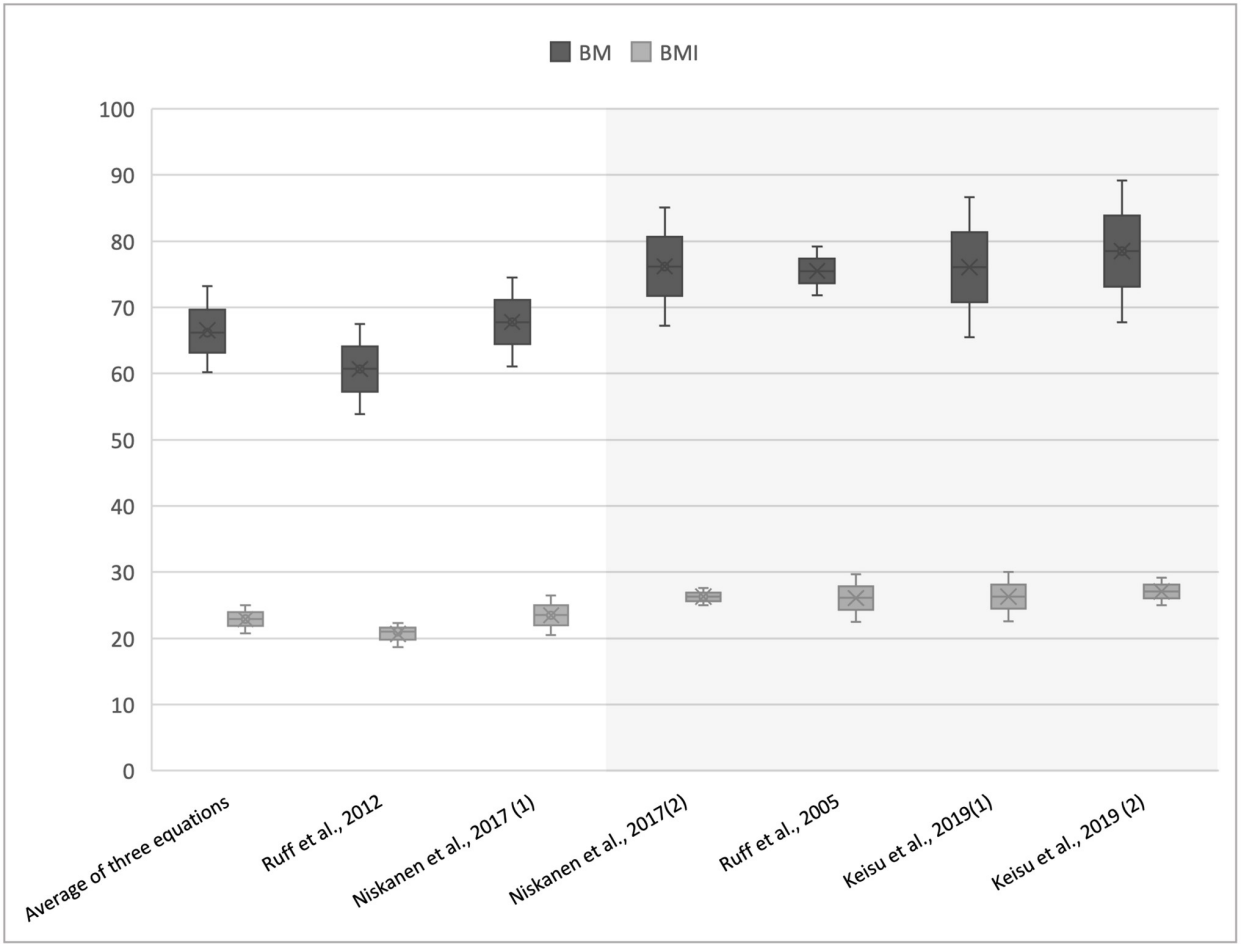
Body mass and body mass index of St Anthony yielded with different regression equations. In the grey box the peak of BM and BMI calculated using knee joint size and ST/BIB equations.

BMI calculations based on the morphometric equations [14, 18] and knee joint formulae suggest a condition of overweight. The variation in estimated BM and BMI, depending on the equations used, is illustrated in Fig 2.

## Discussion and conclusions

We attempted to apply body mass estimation equations obtained from the literature to address questions about the real appearance of St Anthony of Padua, one of the most revered Saint of the Catholic Church. His portraits were strongly influenced by information evinced from literary sources, which from time to time referred to a natural or an acquired corpulence (‘dropsy’).

Body mass estimation techniques are associated with a significant error, but several studies have shown the reliability of these methods as powerful tools in the forensic and bio-archaeological field [33, 46, 47]. The error in estimation depends on several factors. Both morphometric and mechanical approaches seem to perform better in case of normal weight or leaner individuals [17, 48]. Moreover, it has been shown that the error is greater when body mass estimation methods are applied to estimate individual BM, compared to the average BM of a population [17, 49, 50]. In addition, several techniques were tested excluding obese individuals (in most cases, people suffering from joint pathologies) [32, 42]. However, possible *bias* can be solved employing the biomechanical method in combination with the morphometric method [18, 47]. Currently, obesity represents an ongoing pandemic, spreading worldwide with the diffusion of “Western” diet and lifestyle [51, 52]. Thus, the application of equations obtained from current populations to archaeological remains may introduce a systemic error. The great majority of individuals from the past were leaner and smaller than currently, due to differences in diet and activity level [38, 46, 53, 54]. Only small groups enjoyed food security in the past [55].

In other words, BM estimates from ancient skeletons should not be considered as exact body masses, also taking in account that the weight of an individual slightly changes with a circadian rhythm. Nevertheless, BM estimation may contribute considerably to the assessment of the individual body size from the skeleton [56].

The application of body mass estimation formulae to the skeletal remains of St Anthony clearly showed two distinct conditions, from normal weight to overweight, as indicated by the associated BMI (Table 2 and Fig 2). The lowest body mass values derived from the femoral head equation [12, 18, 20, 22, 42] and the three combined equations account for a weight’s range of 60.2± 6.8 to 67.8± 6.7kg. In contrast, formulae based on knee breadth and bi-iliac breadth yielded values of 76.1±10.6 and 78.5±10.7kg [43], and 75.5±3.7 and 76.2±8.9 [14] (Fig 2).

An explanation for these apparently contrasting results might be found in the observation that the femoral head size is more highly correlated with the body weight of an individual at the age of 18 years [12], whereas bi-iliac breadth and development of the associated soft tissues considerably increase in males up to the 40^th^ year of age (0.8 mm/year) [57]. This finding could suggest that the maximum weight of the Saint, reached before his death at the age of 38 years, was between 75.5±3.7 and 76.2±8.93, and his BMI 26.1±3.6 and 26.3±1.3. A strong correlation between the actual body mass and the body mass estimated from stature and bi-iliac breadth has been previously demonstrated, suggesting that this method is more accurate than others to estimate the actual weight at death especially for young individuals and young adults [18, 58].

The response of articular joint size to weight is controversial. Several studies suggest that joint size and shape do not change in response to variations in body mass [30–32]. However, in the study of Keisu et al. (2019) [43], the correlation between knee breadth and body mass at the age of 31 years was high, especially in males, who reach their peak height and physical maturity later than females [59]. According to previous research, the body mass estimated by knee joint size reflects the BM between 18 and 31 years [60]. All things considered, St Anthony might have had a weight between 60.7 ± 6.8 and 67.8 ± 6.7 kg (BMI between 21.0 ± 2.3 and 23.5 ± 3.0) at the age of maturity, should have reached a weight between 76.1 ± 10.6 kg and 78.5±10.7 (BMI 26.3 ± 3.7 and 27.1 ±2.1) at 18–31 years of age, and a weight between 75.5±3.7 and 76.2±8.93 kg (BMI between 26.1±3.6 and 26.3±1.3) at the time of death (38 years).

Following the indications of CDC and WHO, the BMI of St Anthony seems to be in the range of normal weight (BMI 18.5–24.9) at maturity, and in overweight condition (BMI 25 to 29.9) later in life. Currently, BMI classification is widely used in population-based studies to assess mortality risk and can be used as a proxy to assess health conditions in past populations [61]. However, estimating an individual BMI from the skeleton is difficult since it depends on how accurate the estimation of BM and stature is [14]. In addition, large secular increases in rates of growth (weight and stature) have occurred from last century, and the current BMI categories may not be appropriate for individuals of the past [41, 62]. Nevertheless, our estimations seem to be in agreement with the historical and hagiographic information.

Certainly, the weight of St Anthony fluctuated during his life. St Anthony’s life was very active, he went preaching in many parts of Italy, including the hermitage of Montepaolo, and he was in France to fund a hermitage and to preach [63]. He lived with other friars, devoted to praying. Indeed, X-ray examinations showed an enlarged left tibia with marginal osteophyte on the intra-epithelial eminence that demonstrates intense physical activity and long periods spent on his knees [10]. Further, the tibia showed eburnated thickening of the diaphyseal cortex, disappearance of the bony structure and partially obliterated medullary canal [10]. The anthropologists who directly analysed the skeleton of the Saint referred these additional pathological features to non-suppurative osteomyelitis, or sclerosing non-suppurative osteitis, or localised sclerosis of the long bone, or chronic osteomyelitis, which had healed by the time of his death. Written sources report that the Saint was struck in 1220, at the age of 25, by an unspecified “fever” that arose during a period spent in Morocco [2, 4, 6]. After this feverish episode, the Saint’s conditions are described as critical with physical weakness and difficulty in standing [6]. These symptoms might be tentatively linked with the onset of osteomyelitis which had in any case resolved by the time of his death.

In certain periods of his life, we know that he retired to a hermitical life [10], and used to fast from time to time, or only eat bread and water (*Vita prima o Assidua*, XIII, 1-5pp. 160–162). Indeed, his poor eating condition might be confirmed by pathological evidence of *cribra orbitalia* on the skull [10], considered manifestations of anaemic conditions (e.g., [64]). The occurrence of *cribra orbitalia* (grade 3, after Hengen 1971 [65], without no evidence of healing) was assessed by the palaeopathologists Gino Fornaciari, Francesco Mallegni, and Giorgio Raglini. This condition may be due to a diet lacking fundamental elements and malnutrition. The absence of maxillary and mid-facial bones hypertrophy, porosity of long bones, spine deformity, malocclusion, typical signs of thalassemia or sickle-cell anaemia, makes the diagnosis of sideropenic anaemia more likely than a form of Mediterranean anaemia [10, 66].

According to ancient sources, his dropsy was linked to the hermitic life, due to dietary disorders and nutritional insufficiencies [67], while the palaeopathologists who examined the skeletal remains of St Anthony have proposed an unspecified causal relationship of dropsy with the afore mentioned feverish episode at young age [10].

In modern medicine, dropsy could account for a broad spectrum of diseases identified by oedema and ascites, due to accumulation of serous fluids in body cavities [68, 69]. One of the most severe form of dropsy is ascites, a pathological accumulation of fluid in the peritoneal cavity, which might be related to congestive heart failure, an illness, which has been often proposed as the actual cause of the death of the Saint [68, 70–72].

The *Raymundina* also reports about St Anthony’s continuous need for water when he retired to Montepaolo, at the age of 27. Increased thirst in people with heart failure has also been described in connection with dropsy as ’thirsty dropsy’ by past and present physicians [69]. Both *Raymundina* and De Azevedo’s biography mention dropsy only toward the end of his life, and they also refer to an episode of suffocation in sleep that occurred during the Holy Week 1231 and was attributed to the devil’s intervention [4, 6]. In fact, it may have been an attack of dyspnoea, a symptom that could be related to heart failure as well [72]. Patients with a diagnosis of heart failure may notice an unexpected weight gain of >2 kg in 3 days, due to fluid retention [73, 74], but this weight gain is too rapid to be recorded in the skeleton. Possibly, for St Anthony the pathological condition had become chronic and worsened in the last months of his life, since the skeletal analysis of the Saint revealed the presence of an eversion of the last ribs. The index of internal rib curvature suggests a decrease of curvature from the eighth to the tenth rib, probably due to the progressive increase of the intra-abdominal content [10, 75].

In conclusion, in this study we aimed to reconstruct St Anthony’s body mass, define his nutritional status, and find a solution for the riddle of his build, as proposed by the discordant portraits of the Saint. From our analysis of the osteometric measures taken during the ostension of his skeleton, we have proposed that Saint Anthony’s weight was normal at the end of the growing process, while it was higher during the adult age when the Saint reached a condition of overweight. At an uncertain moment in his life, the Saint developed a dropsy–likely due to chronic and progressive heart failure, which contributed to the development of his bloated abdomen. This condition could have worsened with time, becoming a severe form of ascites in the last months of his life.

Iconographic and literary sources emphasised the body size of the Saint as “corpulent” because he was probably overweight, an unusual trait for a preacher of that time. However, the sources likely described the Saint as corpulent because he was also swollen from dropsy. Historical records through ’devotional’ sources have possibly focused on his dropsy to demonstrate the efforts and strength of his spirit, despite his ailing body.

## Supporting information

S1 File (DOCX)
