# Supplementary material for: Belly fat or bloating? New insights into the physical appearance of St Anthony of Padua
Source: PLoS One. 2021 Dec 21;16(12):e0260505. doi: 10.1371/journal.pone.0260505 (PMC8691610; doi:10.1371/journal.pone.0260505)
Supplement: S1 File — (DOCX) [file pone.0260505.s001.docx]

**Supporting information**

**Belly Fat or Bloating?**

**New Insights into the Physical Appearance of St Anthony of Padua**

Jessica Mongillo^1^*, Giulia Vescovo^1,^ Barbara Bramanti^1^

^1^ Department of Environmental and Prevention Sciences, University of Ferrara c.so Ercole I d'Este n.32, Ferrara (Italy)

* Corresponding author

E-mail: jessica.mongillo@unife.it

Table 1. Measurements of maximum lengths of the left femur, tibia, fibula, humerus and the right radius and ulna employed for estimating the stature. The measurements were carried out by Cleto Corrain [10].

|  | Length (cm) |
| --- | --- |
| Humerus (max) | 33 |
| Right Radius (max) | 24.85 |
| Right Ulna (max) | 27 |
| Femur (max) | 47.1 |
| Tibia (total) | 37.8 |
| Fibula (max) | 38.5 |

|  | Length (cm) | |
| --- | --- | --- |
| Femoral head breath (FHB) | *45.5* | |
| Bi-iliac breath (BIB) | 29 | Living bi-iliac (LBIB) 30.93 |
| Femoral bi-epicondylar breath (FBEB) | *86* | |
| Tibial plateau medio lateral (TPML) | 81 | |

Table 2. Measurements of skeletal elements employed for estimating the body mass. The measurements were carried out by Cleto Corrain [10].
